# Cooperative Adaptive Responses in Gene Regulatory Networks with Many Degrees of Freedom

**DOI:** 10.1371/journal.pcbi.1003001

**Published:** 2013-04-04

**Authors:** Masayo Inoue, Kunihiko Kaneko

**Affiliations:** 1 Cybermedia Center, Osaka University, Toyonaka, Japan; 2 Research Center for Complex Systems Biology and Department of Basic Science, Graduate School of Arts and Sciences, University of Tokyo, Tokyo, Japan; University of Basel, Switzerland

## Abstract

Cells generally adapt to environmental changes by first exhibiting an immediate response and then gradually returning to their original state to achieve homeostasis. Although simple network motifs consisting of a few genes have been shown to exhibit such adaptive dynamics, they do not reflect the complexity of real cells, where the expression of a large number of genes activates or represses other genes, permitting adaptive behaviors. Here, we investigated the responses of gene regulatory networks containing many genes that have undergone numerical evolution to achieve high fitness due to the adaptive response of only a single target gene; this single target gene responds to changes in external inputs and later returns to basal levels. Despite setting a single target, most genes showed adaptive responses after evolution. Such adaptive dynamics were not due to common motifs within a few genes; even without such motifs, almost all genes showed adaptation, albeit sometimes partial adaptation, in the sense that expression levels did not always return to original levels. The genes split into two groups: genes in the first group exhibited an initial increase in expression and then returned to basal levels, while genes in the second group exhibited the opposite changes in expression. From this model, genes in the first group received positive input from other genes within the first group, but negative input from genes in the second group, and vice versa. Thus, the adaptation dynamics of genes from both groups were consolidated. This cooperative adaptive behavior was commonly observed if the number of genes involved was larger than the order of ten. These results have implications in the collective responses of gene expression networks in microarray measurements of yeast *Saccharomyces cerevisiae* and the significance to the biological homeostasis of systems with many components.

## Author Summary

Homeostasis is an inherent property of biological systems, which have a general tendency to adapt, i.e., to recover their original state following environmental changes. In cells, this adaptation is mediated by changes in protein expression. Initially, cells respond to environmental changes by altered gene/protein expression; subsequently, the expression of most genes returns to basal levels, albeit not completely, as shown by recent experimental analyses of yeast. Although simple mechanisms for adaptation through network motifs, composed of just a few genes, are well understood, how regulatory networks involving many genes that activate or repress each other can generate adaptive behaviors is unclear. Here, by numerically evolving gene regulatory networks, we obtained a class of genes whose expression dynamics showed adaptation over almost all genes, from which we revealed the general logic underlying such adaptive dynamics with many degrees of freedom, which was not reducible to motifs with a few genes. This adaptation was cooperative, i.e., adaptation of one gene mutually relied upon others' adaptive expressions. Moreover, this collective behavior was robust to noise and mutations. The present study sheds a light on the nature of collective gene expression dynamics allowing for biological homeostasis.

## Introduction

Adaptive responses to environmental changes are fundamental to all living organisms. When environmental conditions change, the cellular concentrations of some chemicals change immediately in response; however, the degree of change is later reduced, returning closer to the basal state. Thus, in general, some variables within a biological system first change in response to environmental changes, but then slowly revert back to pre-stimulus values by adjusting the expression levels of proteins or mediating cellular activity for adaptation to the new conditions. In such an adaptive response, some internal variables change according to the external conditions, while other variables return to the original values, thus realizing both responsiveness and homeostasis.

Recently, simple reaction dynamics models for adaptive response with few degrees of freedom have been studied. For example, Francois and Siggia noted two characteristics in such responses: responsiveness and perfectness of adaptation [1]. They carried out numerical simulations of the evolution of parameter values in simple network motifs consisting of three components to show that both these characteristics are realized. Similarly, Ma et al. studied all possible three-node enzyme network topologies numerically to identify those that exhibit adaptive responses [2]. They found that only two major core topologies can show an adaptive response: a negative feedback loop with a buffering node and an incoherent feed-forward loop with a proportioned node.

In fact, such adaptive responses have been studied with simple chemical reaction models with a few components (e.g., proteins) [3]–[8]. After the immediate response, the expression of one component returns to its original value, and changes in the external conditions are compensated for by adjusting the other components within the system. Such simple chemical reaction dynamics are also abstracted from complex reaction networks as motifs, as mentioned above [9], [10]. However, in real biological reactions, the expression levels of many proteins influence each other through mutual activation and inhibition of gene expression. Adaptive responses stemming from such complex reaction dynamics involve a huge number of chemical species or the expression dynamics of many genes. Indeed, the simple network motifs proposed above may exist as a part of a network but cannot function in isolation [11]. Although simple models could possibly be derived by reducing the degrees of freedom in a complex reaction network, no such reduction scheme is yet available. Therefore, it is important to study adaptive responses within a system consisting of many proteins.

One reported example of such an adaptive response with many degrees of freedom concerns the gene expression patterns in yeast *Saccharomyces cerevisiae* subjected to diverse environmental changes, including temperature shock, hydrogen peroxide treatment, amino acid starvation, and nitrogen source depletion. Studies using DNA microarrays have shown that certain sets of genes (approximately 900 genes) exhibit similar responses to almost all of these environmental changes, while some genes show unique response patterns to specific conditions only [12]–[14]. For example, after a temperature shift, many genes are either up-regulated or down-regulated shortly after the stimulus and then gradually return to pre-stimulus expression levels. Moreover, many genes that do not specifically respond to heat shock stimulus also show adaptive responses. Such responses are called “stereotyped” responses, involved in protecting and maintaining critical features of the intracellular system. Several other reports have also suggested that a large fraction of genes, i.e., approximately 50%–70% of genes, show adaptive responses. The response is not monotonic; initially, genes expression is altered in response to the stimulus, but this change is later compensated for, at least partially. That is, many gene undergo initial up-regulation followed by down-regulated, or vice versa, returning to (nearly) basal expression [15], [16]. Such adaptation without complete return to the original level is termed as partial adaptation.

This type of multidimensional adaptive behavior should be quite rare in an arbitrary dynamical system with many degrees of freedom. Indeed, it is not common for a system to exhibit changes in a large number of variables in response to input parameters (environmental conditions) and later return these variables to the original values. Furthermore, designing such a system would become increasingly more difficult as the number of involved variables increases. Unveiling the characteristic properties of such an unusual dynamical system of gene expression is the main purpose of the present study.

If such singular behavior is observed ubiquitously in biological systems, one possible origin could be selection through evolution. That is, through the selection of functional networks for higher fitness values, rare networks exhibiting such atypical behavior may evolve. Here, however, we should note that the existence of such selective pressure toward adaptive dynamics over the expression of many proteins is not easy to imagine. For a given environmental change, selection process to achieve the adaptive response of only one or a few specific genes would be naturally expected. There is no need to postulate that many genes exhibit adaptive expression responses for a fitness for selection. Hence, it is important to determine how genes that do not need to be adaptive indeed show adaptive response collectively, as is commonly observed in responses of micro-organisms. Can such adaptive responses over many genes evolve through a numerical evolutionary process of gene regulatory networks by imposing a single, simple fitness condition?

Here, we answer this question by examining the evolution of regulatory networks involving changes in the expression levels of many genes. We numerically evolved these networks by using genetic algorithms with a fitness condition for the adaptive behavior of only a single target gene. Although the specific evolutionary course may not be realistic due to the simplified fitness conditions adopted here, ‘cooperative’ adaptive responses were generally observed. Hence, we expect that these shed a new light on characteristic features of adaptive systems. We also discuss the relevance of such adaptive responses over many genes, cooperative in nature, to biological functions and the possible relationships of these responses with gene expression patterns in yeast *Saccharomyces cerevisiae* observed by microarray analysis.

## Models

### Gene regulatory network model

We modeled gene expression dynamics using a regulatory network to study adaptive response with many degrees of freedom, following the methods presented in earlier studies [17]–[21]. In a regulatory network, there are 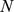 nodes corresponding to each gene. The expression level of a gene is represented by the variable 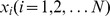. By appropriately normalizing gene expression levels we set 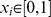, where 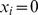 represents a suppressed state and 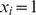 represents a highly expressed state.

#### Gene expression dynamics

**Figure 1:**
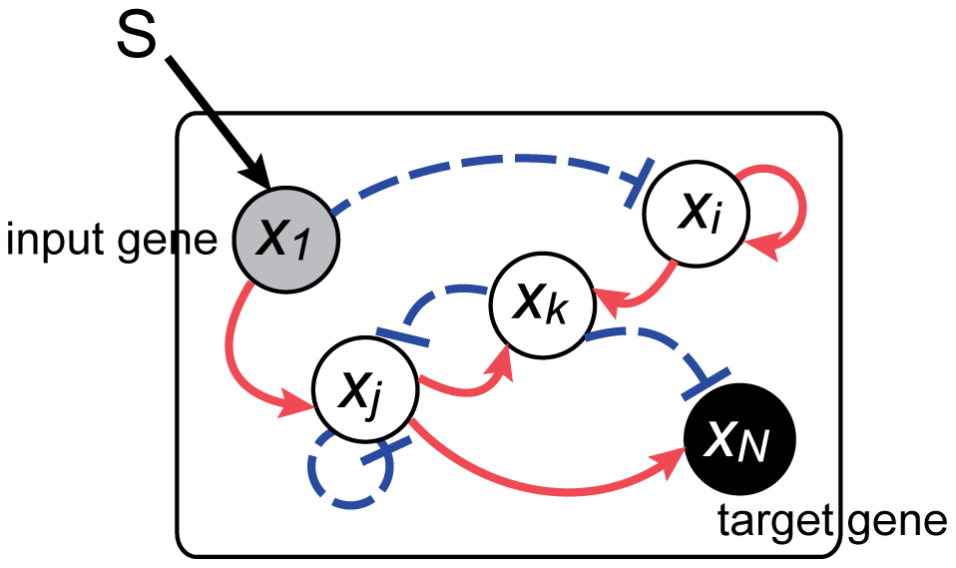
Schematic view of the reaction process in our gene regulatory network model. Each circle represents a gene whose expression level is denoted by 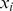. Interactions between genes are shown by arrows; arrows with solid red lines show activation whereas arrows with broken blue lines show suppression. The input (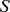) acts on the input gene and influences the response of the target gene through these interactions.

By assuming that the synthesis and degradation of mRNA is much faster than protein synthesis, the concentration of mRNA is adiabatically eliminated [22], such that the protein expression level is proportional to the mRNA concentration (gene expression level). Thus, gene expression was not distinguished from protein expression throughout this paper. The expression level of a gene (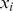) is regulated through interactions with other proteins, thus constructing a gene regulation network, and the expression of each gene can change with time. We defined input genes, which receive the external signal (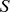), and target genes, which are responsible for the output behavior and determine the fitness or function of the network. For simplicity, we considered the simplest case with a single input gene and a single target gene. Without loss of generality, we assigned the input gene to gene 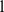 and the target gene to gene 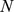 (Figure 1). Each gene interacts with others by activating or suppressing gene expression. In this regulatory network, the applied external signal (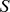) is transmitted from the input gene to others, and through mutual regulation, the external signal ultimately influences the activity of the target gene.

The above gene expression dynamics are described by a gene regulatory network. Here, we adopted the following equation to describe the time evolution of expression levels (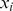).
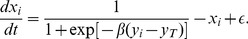
(1)The first term represents interactions with other genes, where
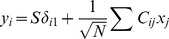
(2)with 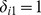 (for 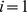), 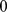 (for 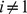) and 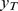 as a constant threshold for expression. Here, 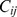 represents regulation from gene (protein) 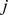 to 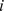, and the elements of the regulation matrix 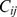 are 0, 1, or −1 depending on whether the interaction is non-existent, excitatory, or inhibitory. The interaction term with other genes was scaled with 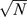. We adopted this scaling to certify that 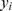 took a value of a comparable order with 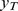 regardless of 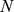 (if the distribution of signs of inputs to each gene was not biased). Furthermore, we prohibited feedback interactions from the target gene, i.e. 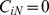. This condition was set to eliminate the possibility that the adaptive response of the target gene forced other genes to behave adaptively. (Note, however, that the results do not change essentially even if 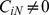). As 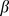 increases, the first term in eq.(1) approaches a step function with a threshold 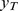. Gene 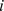 is active only when 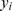 exceeds the threshold value. The second term represents degradation, while 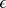 is a small output representing spontaneous expression levels. With this addition, the maximal expression level is shifted from 1 to 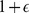, so that 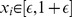. However, this does not affect the results as long as 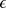 is sufficiently small, say 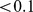.

In the following study, we set the parameter values to 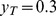, 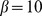, and 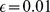, unless otherwise mentioned, and the results did not change as long as these values were in an appropriate range (i.e., 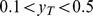, 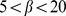 and a small positive value for 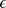). Dependence on 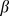 will be discussed in the Results section.

Eq.(1) is a simplified gene regulatory network model and has been discussed extensively [17]–[19], and the evolution of such a gene regulatory network has been simulated in previous studies [20], [21]. This is a simplified model of gene (protein) expression dynamics with an external input. However, it has the potential for adaptive dynamics upon input change, and captures mutual activation and inhibition among gene products, which constitute complex networks over many genes. By using a simple example for such system, we expect to extract generic features of adaptive dynamics with many components, which will be valid even for a system in which details are modified to more closely match the conditions of real cellular systems. In the following discussion, we evolved the regulation matrix only and kept other parameters constant.

#### External signal from environment

As shown in eq.(2), the external input is applied only to gene 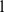, where the term 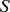 is set at 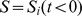, and is then switched to 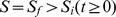. Following the application of the external signal (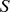), the expression of genes shows a temporal response through mutual regulation. We studied the temporal response from the steady state under 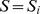 to the new steady state under 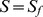. The expression levels of all genes are set to 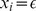 as an initial state and are then evolved with time according to eq.(1), with 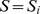, until expression reaches a steady state (fixed point). Then, the external input is switched to 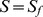 at 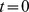. Here the initial condition for 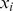 was set so that all genes are in off state as this initial condition is the most difficult one for cooperative adaptive response to evolve. Moreover, we supposed 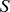 shifts from no signal to a sufficiently large value so that the input gene can respond to 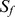 even with disturbing input from other genes. Unless otherwise mentioned, we fixed 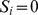 and 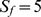 throughout the paper; the results were not affected by the change in these specific values as long as the 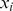 initial value and 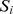 is enough small compared with 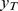 and 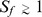.

### Selection process with genetic algorithm

Next, we evolved the regulation matrix by imposing the fitness condition that the target gene shows an adaptive response.

#### Fitness function

**Figure 2:**
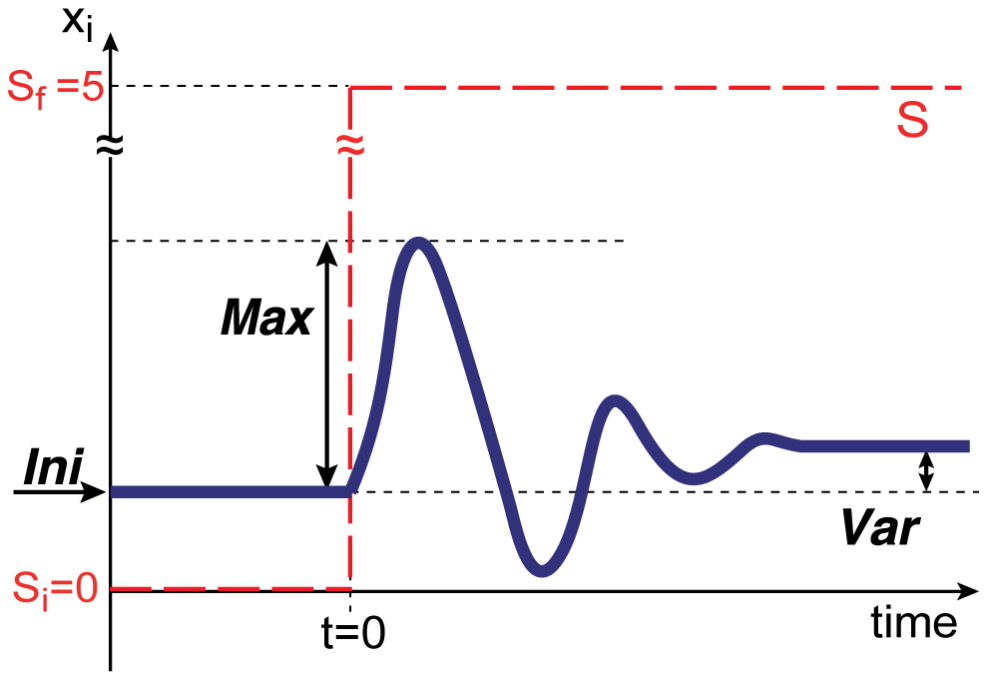
Definition of our evaluation function. Three elements, 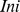, 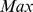, and 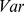, were defined as shown. The input (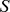) is shown by the broken red line, and the output is shown by the solid blue line. 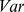 was defined as an average value over some duration.

For the adaptive response, we postulated the following two properties (Figure 2). First, gene expression exhibits a large response immediately upon a change in the signal 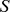. Second, its steady-state activity does not depend on the 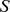 value. These conditions are quantitatively characterized by introducing 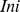, 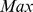, and 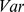 as follows. 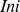 is the steady-state activity before the application of the signal switch at 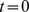; 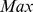 is the maximum change in the response from the 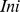 value (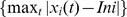); and 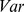 is the deviation of the steady-state activity from the 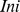 value, i.e., 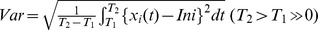. We defined 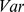 as the time average since gene expression levels may show temporal oscillation (with a small amplitude) at the final steady state. These two postulates are characterized by large values of 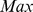 and small values of 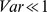. We designed the evolutionary process to optimize these two properties by defining 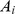 as the difference between the maximum response value and the deviation of the steady-state activity of gene 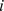.
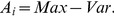
(3)The fitness function is given as this 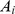 value for the single target gene (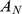). Note that a similar fitness function was adopted by Francois and Siggia [1] when they evolved a reaction network with three elements. With this fitness function, there will be a selection pressure to maximize the response peak (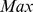) and minimize deviations in the steady-state activity (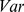). The maximal value of this fitness function is 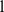 because the expression level is limited to 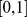 (or precisely 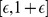) such that 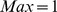 and 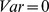 at best.

According to this fitness function, fitness is maximized by the perfect adaptation of the target gene (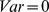). Such adaptation implies restoration of the original state, as expected from homeostasis [3]. Of course, perfect adaptation of expressions of many genes would be too strong of a demand; however, the demand only to some life-threatening states (gene expression) may not be absurd. This simple choice of the fitness function is sufficient for our study on adaptive expression dynamics of many genes.

#### Selection procedure

In the present paper, we used a simple genetic algorithm to evolve the network structure, i.e., the interaction matrix. For each generation, we prepared 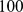 networks containing 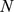 genes each. After applying a signal of magnitude 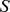 to the input gene, we calculated the fitness of each network using eq. (3). Among the networks, we selected 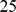 with the highest fitness and discarded the others. Then, from each selected network, 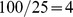 mutant networks were generated, i.e. the selection process did not include noise. In the mutation process, elements 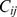 are randomly selected with a given mutation rate 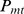 (which is small), and their values are changed to one of the other two values among 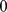 and 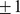. The 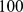 newly generated networks constituted a new generation, and the selection process was repeated again.

Here, we first presented the results for 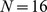 and subsequently discussed 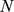-dependence. We evolved only the network structure and kept other parameters unchanged in order to study the importance of gene interactions in the evolution of the adaptive response of the target gene in a system with many degrees of freedom, in contrast to the previous studies [1], [2], where the parameter values are tuned for minimal gene regulation networks to achieve the adaptive response. By focusing only on the network structure, we globally scanned the expression dynamics with a huge variety of networks with many components.

## Results

### Evolution toward cooperative adaptive responses

**Figure 3:**
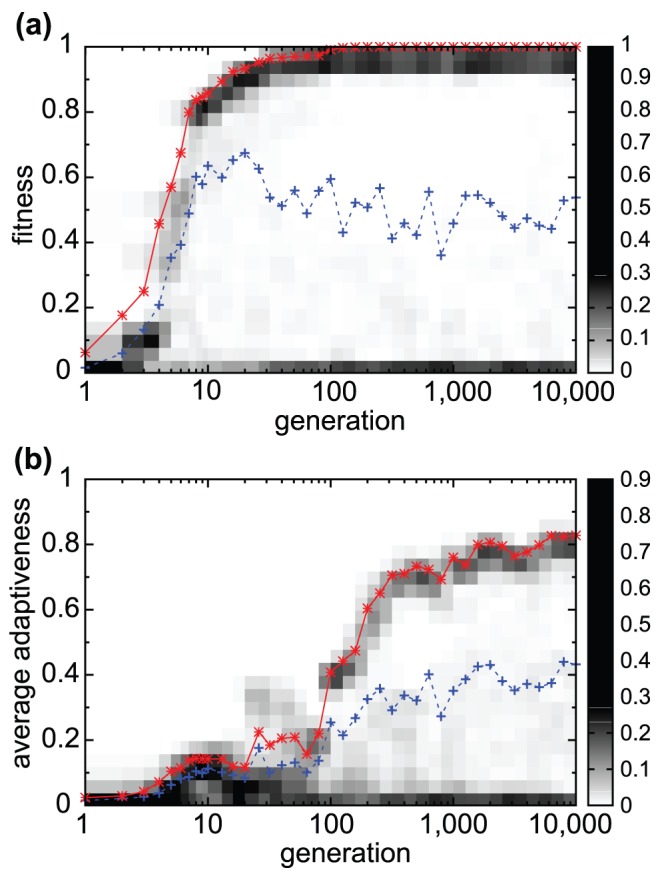
Evolution towards cooperative adaptive responses. Changes in fitness values (a) and average adaptiveness 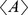 (b) over generations in the evolutionary course. Generation numbers are shown in the 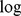 scale (abscissa). Population density distributions of fitness values (a) and average adaptiveness (b) at each generation are plotted in pixels (gray scale) according to the bar on the right side. Hence, for later generations, the distribution peaks at a high fitness (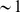) and high average adaptiveness, while another peak exists at almost zero fitness and zero average adaptiveness. The average values over surviving individuals (solid red line) and over all of the networks (dotted blue line) are also plotted. Networks with the highest (or lowest) fitness correspond to those with higher (or lower) average adaptiveness. 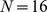, 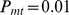, 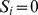, 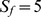, 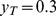, 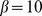, and 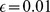. In the following figures, these parameters are adopted unless otherwise mentioned.

In our model, the adaptive response of the target gene evolved within a few hundred generations to the highest fitness (Figure 3(a)). Here, fitter networks that produce offspring reach almost the highest fitness value after evolution, whereas the minimal fitness value of the networks that are discarded are 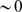.

**Figure 4:**
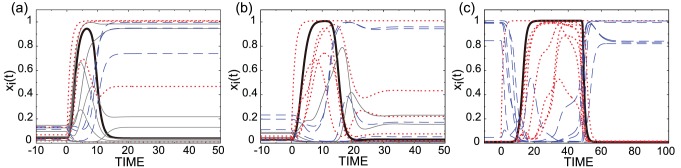
Evolution of expression dynamics toward cooperative adaptive responses. Temporal changes in the expression levels of all genes in the network with the largest fitness value for a generation in each stage: (a) 10th, (b) 80th, and (c) 1,000th generation for 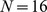 and 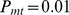. An 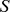 shift was added at 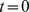. The response of the target gene is shown with a bold black line. The expression levels of target-activating genes are shown with dotted red lines, those of target-inhibiting genes are shown with dashed blue lines, and genes having no interactions with the target are shown with thin straight gray lines. The plateau in the maximum response of the target is commonly observed in networks with large average adaptiveness, albeit it is not requested by the fitness function itself. Note the differences in scales of time (abscissa). (a) fitness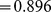, 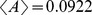; (b) fitness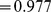, 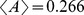; (c) fitness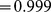, 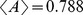.

Now, we investigate the response of non-target genes. The response of each gene expression (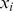) to a change in 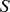 is generally classified into the following three cases: (i) perfect or partial adaptation, (ii) monotonic (non-adaptive) relaxation, and (iii) no response (no change at all). Under the fitness conditions set here, the responses of non-target genes do not matter at all, and they can be either adaptive, monotonic, or non-responsive. As seen in Figure 4, however, the responses of an increasingly larger number of genes turn from monotonic to adaptive with continued evolution. In the early generations (Figure 4(a)), almost all non-target genes show monotonic relaxation, but with evolution, more genes with monotonic responses begin to show adaptive responses (Figure 4(c)). To characterize this trend quantitatively, we measured the ‘average adaptiveness’, i.e., the average value of 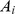, defined by 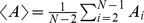 for 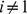 and 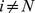. We did not include the input and target genes for the average, since the input gene always shows monotonic increase, while the target gene always shows adaptive behavior after generations to achieve a high fitness value. The average adaptiveness 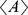 does not always tightly correspond to the fitness value and there are some networks with high fitness values and small 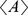. However, through the course of evolution, as shown in Figure 3(b), we see that an increase in the fitness value is accompanied by an increase in 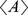, and networks with larger 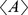 have higher fitness values in general. By comparing Figure 3(a) with (b), it can be seen that the fitness first increases to a relatively high level, and then the increase in average adaptiveness progresses with generations while the fitness of the target gene keeps on increasing, albeit gradually. After the first increase in fitness, many tiny increases are achieved resulting in large 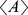. We term such response with adaptive responses by many non-target genes, i.e. with large 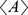 value, as a ‘cooperative adaptive response’. Note that in this model, as we prohibit feedback interactions from the target gene, i.e. 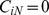, the adaptive responses in non-target genes do not originate in the adaptive response of the target gene. Moreover, from the fitness condition itself, there is no selection pressure for the adaptive responses of non-target genes. Perfect adaptation of the target gene is achieved rapidly, and the cooperative adaptive responses of the non-target genes evolve subsequently though there is no request.

### Evolutionary changes in gene expression dynamics

Now we take a closer look at the high-dimensional gene expression dynamics involved in realizing this cooperative adaptive response. The dynamics show three stages of evolutionary change in reaching this cooperative adaptive response.

#### (i) Timing difference

At the first stage (Figure 4(a)), a timing difference strategy evolves among genes showing monotonic responses: Almost all genes that influence the target gene show a monotonic increase from 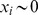. Those genes that activate the target gene show an earlier increase, and those that inhibit the target gene show a later increase. By exploiting this timing difference, the target gene is first activated and later suppressed to realize an adaptive response.

#### (ii) Adaptive response in target-activating genes

At the second stage (Figure 4(b)), genes with adaptive response appear but only among those that activate the target gene. At this stage, the target gene is still inhibited by genes showing monotonic increase with a time delay. The initial expressions of both genes that activate and inhibit the target gene are at around 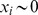. Here, the first mechanism with the time delay remains, but because the target-activating genes develop an adaptive response, the return of the target gene expression to 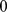 is strengthened.

#### (iii) Cooperative adaptive response

At the final stage (Figure 4(c)), a cooperative adaptive response is achieved for almost all genes that directly influence the target. Generally, the target gene shows upward adaptive responses in this model and target-activating genes show upward adaptive response (i.e. the expression level 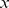 starts from 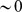, increases upon the application of 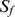, and later decreases), whereas target-inhibiting genes show downward adaptive response (i.e. 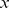 values start from around 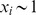, decrease by the application of 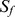, and increase later). As a result, the target gene can show the (upward) adaptive response required by the fitness condition. (In rare instances, downward adaptive responses are realized for the target: the target activating genes show down/up responses and the inhibiting genes show up/down responses in such networks). An increasingly larger number of genes differentiate into either of the above two groups as the average adaptiveness 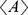 increases, until almost all genes that are neither input nor target belong to either of the two as 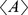 approaches 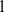. The number of genes that do not have a direct impact on the target gene(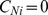) decreases as 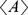 increases. When this cooperative adaptive response achieves a high fitness value, the genes always differentiate into these two groups and genes in each group show contrasting responses.

This cooperative adaptive response is not a stipulation in the definition of the fitness function itself. However, such cooperation enhances the adaptive response of the target gene as follows: For the target gene to obtain a large fitness value, it is necessary to provide an input that is sufficiently larger than the threshold value 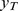 and also subsequently provide strong inhibition. This is obtained from the adaptive signals from other genes, which allow the target to develop an adaptive response. Furthermore, as the number of genes providing these signals increases, the adaptive response is enhanced. In the cooperative adaptive mechanism, almost all genes contribute additively to the adaptive response of the target, either by activation or inhibition, whereas in the timing difference mechanism at the first stage, only the difference between activating and inhibiting genes can contribute to the adaptive response of the target, so that the effective number of genes that contribute is half the total at maximum. In this sense, the cooperative adaptive response can afford higher fitness.

### Network size and motifs

**Figure 5:**
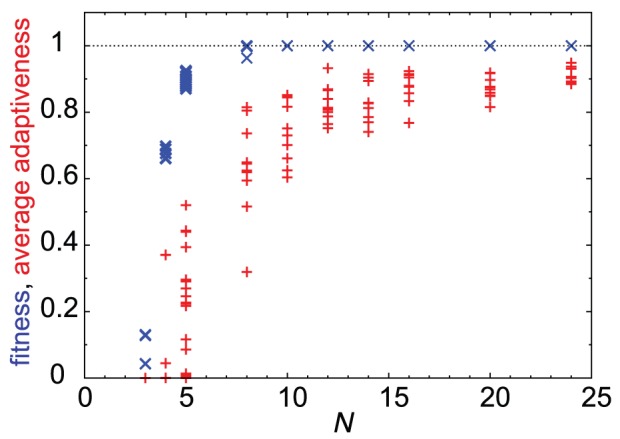
Cooperative adaptive responses can evolve only in a system with many degrees of freedom. Changes in fitness (blue 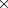) and average adaptiveness (red 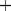) (ordinate) according to 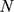 (abscissa). For 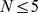, we searched all networks and identified those with high fitness; fitness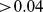 for 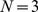, fitness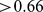 for 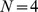, and fitness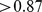 for 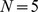. While, for 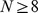, the values for the network with the largest fitness at the 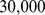
th generation are shown for 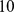 different strains for each 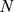. 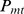 was set to satisfy 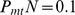 for 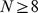.

Cooperative adaptive responses require other genes that show adaptive responses and thus represent the collective dynamics through variations in a number of genes. Hence, this type of response can be expected to require a certain minimum number of genes. In Figure 5, it can be easily observed that the average adaptiveness 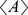 was small for a small network size but increased as the networks became larger. In the region 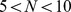, the average adaptiveness were distributed depending on the evolutionary course. However, for 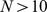, cooperative adaptive responses with a large 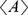 values were always attained. Thus, cooperative adaptive responses require a sufficiently large number of genes, and do not emerge at all for 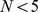.

The fitness values were indeed smaller for 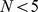, compared with the case for 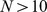 with 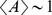. The fitness value increases toward the maximal level as 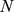 increased from 5 to 10, accompanied by an increase in 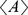. (Note, however, the fitness for 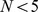 could be increased by fine-tuning the parameters of the gene expression dynamics [1], [2], while such tuning was not necessary for 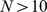). Moreover, networks with larger 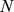 showed higher performance than smaller networks (Figure S1).

**Figure 6:**
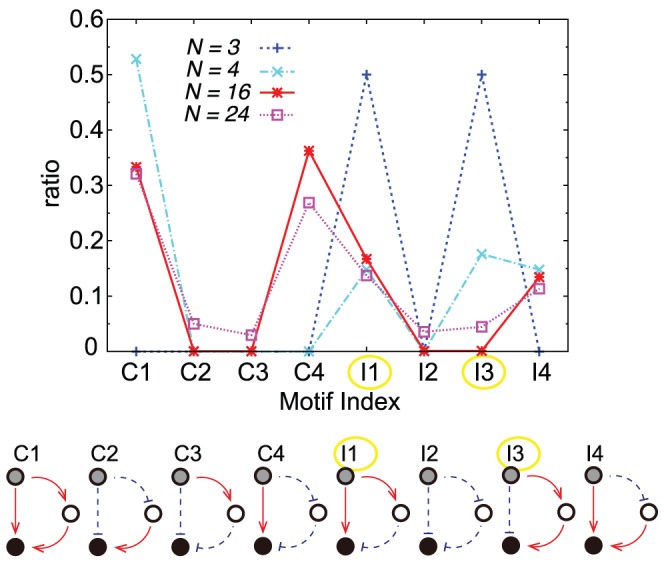
Network motifs in cooperative adaptive networks. Fractions of each feed-forward-loop (FFL)-type network motif are shown. These ratios were counted among the input (gray circle), target (black circle), and middle genes and normalized by the total number of FFL-type network motifs. Networks with fitness 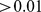 for 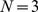 and with fitness 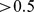 for 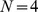 from all possible networks were used. For 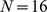 and 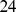, networks satisfying fitness 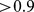 from 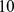 different evolutionary trials were used. 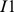 and 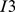 were minimum adaptive motifs, where an arrow with a solid red line indicates activation and an arrow with a broken blue line indicates inhibition. For the case in which 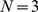, each of these motifs occupies about 50%. For 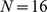 and 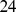, the frequencies of the motifs 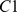 and 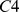 were larger.

**Figure 7:**
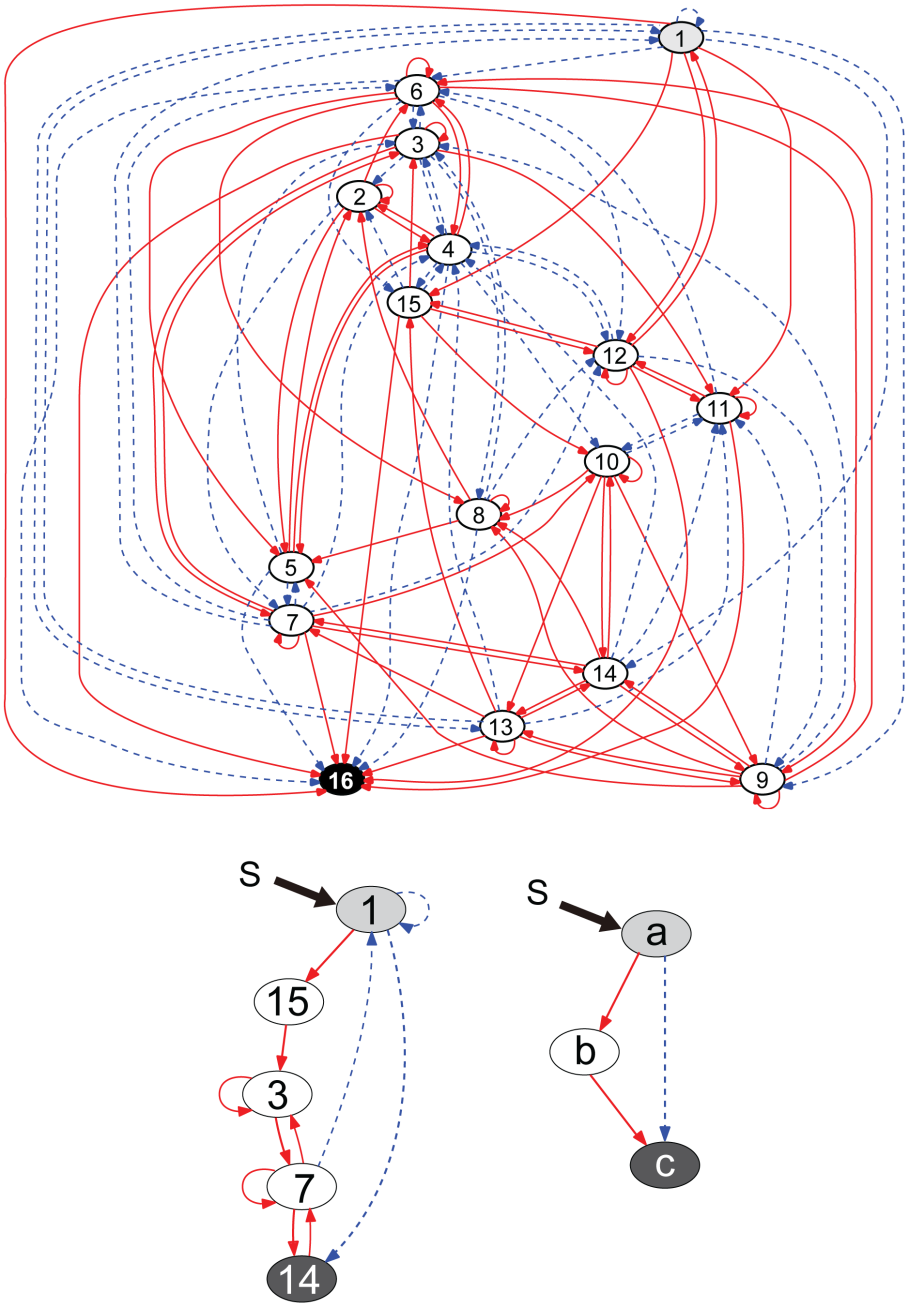
Network structure of a cooperative adaptive network. (upper) An example of cooperative adaptive networks with 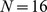 which does not include the minimal adaptive motifs of 3 genes at any part of the gene regulatory network, but shows adaptive behaviors for all genes (except the input gene). The input (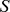) acts on the input gene (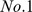) and the target gene is 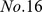. Interactions between genes are shown by arrows; arrows with solid red lines show activation, whereas arrows with broken blue lines show suppression. (lower left panel) An example of a substructure showing a similar role with the minimal adaptive motifs extracted from the upper network. For reference, a related adaptive motif with 3 genes (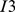 in Figure 6) was redrawn (lower right panel).

Now, we examine the network motifs to achieve the adaptation. According to a previous study [2], only the two types of network motifs with three elements, i.e., the incoherent feed-forward loop (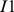 and 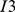 in Figure 6) and negative feedback loop [23] can show adaptive responses. For the feed-forward network 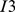, as redrawn in Figure 7 (lower right panel), the gene 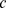 showed a decreasing response by direct negative interaction with input gene 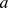 right after the imposition of the input 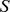, but later increased back to original levels as a result of the positive input of the gene 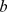, with a time delay. Thus it showed a downward adaptive response.

In a small network with 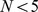, which is noncooperative, such motifs and their combined motifs appear with higher frequencies than others. These play a major role in the adaptive response of the target gene. On the contrary, in a network with many degrees of freedom, these motifs are not frequent. Only at the initial stages of evolution adopting the timing difference strategy, they appear frequently, and the network is reduced to this type of incoherent feed-forward loop when coarse-grained. Later, however, with the increase of average adaptiveness 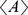, their frequency decreases, and it is much smaller than that expected for a random network.

Moreover, such three-gene motifs for adaptation can be completely deleted while maintaining higher fitness and 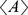 values. In Figure 7, we show an example of these types of networks, which exhibit adaptive behavior with a high 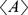 value; these networks did not include any of the three-gene adaptive motifs. Instead, other motifs become more frequent for networks with a high 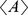 networks (Figure 6 and Figure S2).

We focused on the structures of the input, output, and middle genes that intervene the two. As the negative feedback loop is not so relevant to minimum adaptive motifs in our model, and the frequency of occurrence of such negative-feedback motifs was indeed very small, we focused on the frequencies of the feed-forward loop motifs [24]. We found that the minimal adaptive motifs (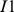 and 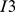 in Figure 6) appeared frequently in small networks, but were less frequent in large networks. Instead, 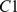 and 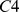, which are not adaptive motifs, become more frequent.

We also studied network motifs with all possible combinations of three genes (Figure S2). We found that minimal network motifs for adaptation with three genes occurred at about the same frequency as random network cases. Moreover, they decreased in frequency as average adaptiveness 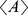 increased. In contrast, motifs exhibiting mutual inhibition and activation were present in a significantly high fraction of cases and increased in frequency with increases in average adaptiveness (Figure S2).

**Figure 8:**
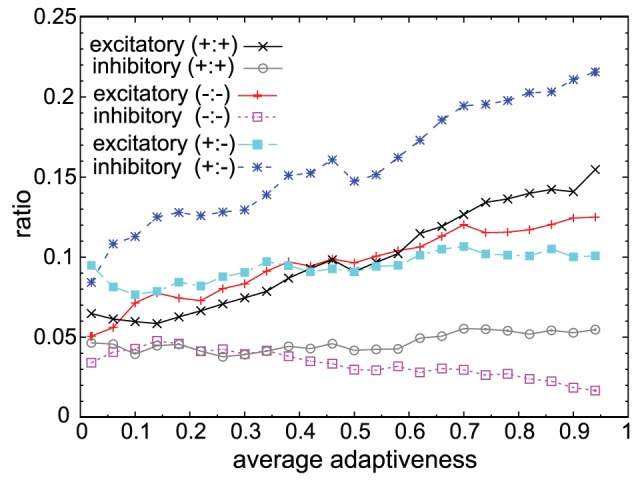
Change in interaction types among or between target activating and inhibiting genes. Fractions of each excitatory/inhibitory interaction between target-activating genes (black cross/gray circle), between target-inhibiting genes(red plus/magenta open square), and between target-activating and inhibiting genes (cyan filled square/blue asterisk). Networks satisfying fitness 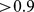 from 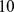 different trials with 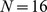 and 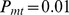 were used. Genes differentiate into two groups, target activating or inhibiting groups, and genes in the same group often activate each other, while genes in different groups inhibit each other mutually. The fractions of such interactions increase as average adaptiveness (abscissa) increases.

As already mentioned, in cooperative adaptation, genes differentiate into two groups, activating or inhibiting, with respect to the target gene. The dominant motifs – 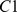 and 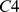 in Figure 6 – indeed corresponded to these two groups. For this separation into two groups, genes in different groups often inhibit each other mutually, while genes in the same group activate each other (Figure 8). That is, genes that provide positive inputs to the target gene activate the target as well as other target-activating genes, repress target-inhibiting genes, and vice versa. Thus, motifs exhibiting mutual inhibition and activation increased in frequency as average adaptiveness 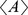 was increased (Figure S2). In summary, networks showing cooperative adaptive responses with many degrees of freedom have characteristic structures different from those of non-cooperative networks with few degrees of freedom.

### Network structures of cooperative adaptive networks

Now we examine how some networks show a ‘cooperative adaptive response’, without standard minimal adaptive motifs of three genes. An example of such a network is shown in Figure 7. Although there is no three-gene motif, such networks generally include structures with four or more genes that play a similar role as minimal adaptive motifs, somewhere in the whole network. In Figure 7 (lower left panel), for example, a core structure from Figure 7 (top) was extracted where genes 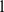 (input gene), 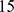, 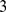, 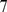, and 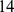 form this type of structure, with a feed forward network. Here, in such extracted motif, we call the gene 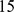, 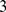, and 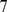 ‘mediator’ which correspond to the gene 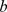 in the three-gene motif (lower left panel of Figure 7), and call the gene 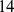 ‘receiver’ which corresponds to the gene 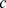. A receiver gene is not necessarily the target gene in the whole network. In this case, the receiver gene 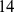 showed a downward adaptive response as a result of direct negative input from gene 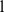, and later positive input mediated by three genes (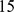, 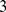, and 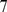) with a sufficient time delay. In essence, a rapid direct input from the ‘input gene’ and an opposing delayed input from the mediator gene(s) comprised the adaptive response.

**Figure 9:**
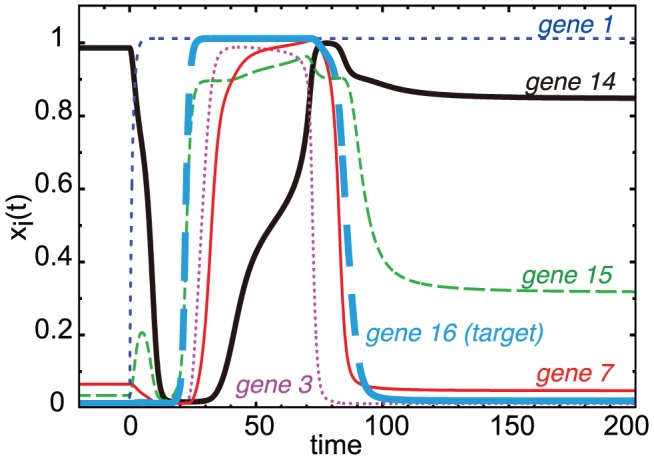
Expression dynamics in cooperative adaptive networks. Responses of some genes that form the substructure shown in Figure 7 (lower left) extracted from the network shown in Figure 7 (top). The time series of 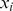 for 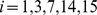 and 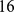 (target gene) is plotted with different colors, after 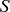 is changed from 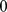 to 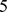 at time 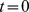. The response of? the receiver (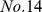) is shown with a solid black bold line. Here, the target gene (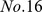, solid cyan broken line) shows perfect adaptation with a plateau at 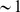 for times between 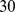 and 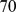.

So far the mechanism for the adaptive response of the receiver gene was common with the minimal adaptive motifs. However, there is a critical difference in the cooperative adaptive response, i.e., mediators show adaptive responses rather than monotone responses. In this case, gene 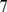 showed an adaptive response, as demonstrated in Figure 9. This is a strong contrast with the minimal adaptive motif, where the mediator genes show monotonic response (as in 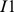 and 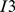 in Figure 6). When mediators show adaptive responses, the receiver is rapidly activated (or inhibited) by the input gene and is later inhibited (or activated) by mediators. This can result in the adaptive behavior of the receiver gene. However, there is some ‘danger’. When the mediators returned to original levels, then the total input to the receiver would also return to its original state (at 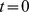) again. Then the up-down (or down-up) response in the receiver expression would be repeated again. Thus, the expression of the receiver would repeat cyclic (oscillatory) response.

In fact, this cyclic response was avoided in the following way. The receiver gene received inputs not only from its direct mediator (gene 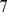 in Figure 7) but also from other genes (gene 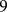, 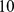, and 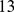), such that the expression of the receiver was not driven by the mediator alone. Before the mediator showed adaptation back to its original expression, inputs from other genes showing partial adaptations settled to levels sufficiently different from the original.

An alternative process to avoid the cyclic response, which was often adopted in some evolved networks, is the partial adaptation of the mediator(s). Then the input to the receiver does not come back to the original value, so that the receiver can be settled to show the adaptive behavior. In general, partial adaptive response of a gene can introduce partial adaptation of other genes, and thus, all genes can show adaptive expression, most of which are not perfect but partial. Indeed, 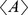 never reached 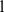, but instead was able to reach 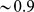 at most, while the fitness could be as high as 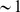, as seen in Figure 5. These partial adaptive responses ensured the adaptive response of the target. Each receiver showed adaptive behaviors, by appropriate degrees of partial adaptation. The degrees of partial adaptation were determined in a self-consistent manner over all genes to achieve their adaptive response.

### Robustness

Next, we investigated the robustness of adaptive behaviors in the context of noise and mutations. In cooperative networks, most genes showed adaptive responses with plateaus at their peak values. One might expect, then, that for such case, the peak value would not be easily changed by perturbations, implying robustness. However, this was not necessarily true for all conditions; indeed, networks with very large 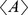 values were not robust to mutations and noise.

**Figure 10:**
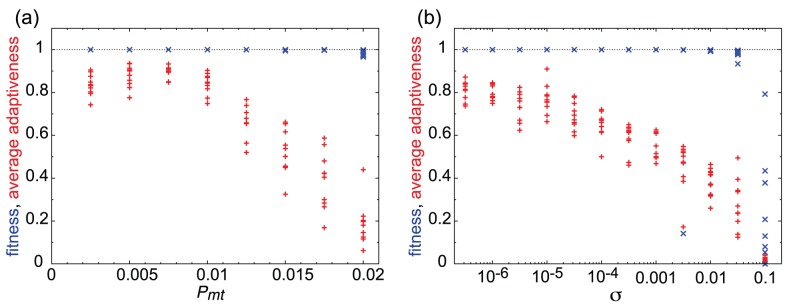
Evolution under large mutation rates and noise. Changes in fitness (blue 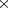) and average adaptiveness (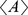; red 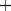) (ordinate) according to (a) the mutation rate 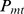 or (b) noise strength 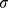 (abscissa). Values of networks with the largest fitness values after a sufficiently long evolutionary duration are shown for 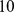 different strains simulated with (a) 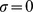 and (b) 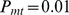 for each. In (b), fitness and average adaptiveness measured without noise were plotted using networks evolved under each noise level.

**Figure 11:**
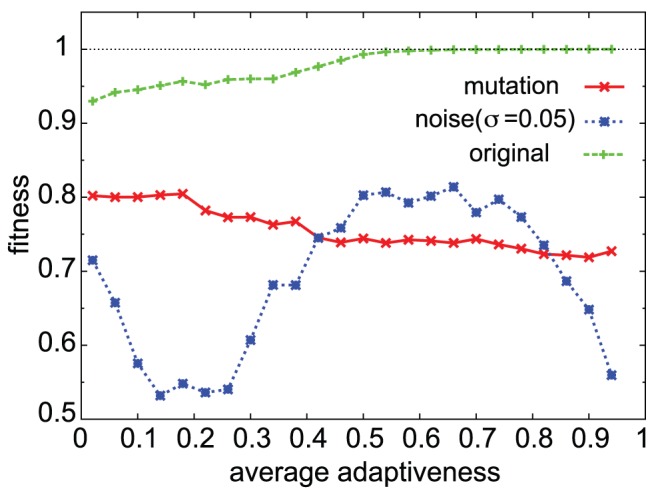
Robustness of cooperative adaptive networks. Changes in the fitness value (ordinate) under mutations (red 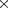) or noise (blue 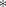), for evolved networks with different average adaptiveness (abscissa). We first evolved networks with 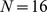, 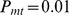, and 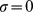, and then sampled those networks with fitness values 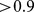. We repeated 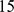 different runs of evolutions to sample such fitted networks, and collected a total of 20,038 networks. We then made a histogram of average adaptiveness values with a bin size of 0.04. For networks in each bin, we removed a single path directly connecting any 2 genes. We computed the fitness values for such emulated networks over all possible removals of single paths. After averaging all networks in the average adaptiveness of a given bin, the average fitness value by mutation was obtained. Also, instead of mutations, we added a noise term in the model as mentioned in the text, with the noise amplitude 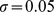, and computed the fitness. Again, by averaging over the networks in a given bin of average adaptiveness, the fitness under noise is obtained. The original fitness values are shown with green 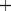 symbols.

To study robustness in the context of mutations, we carried out numerical simulations with increasingly large mutation rates. As shown in the results in Figure 10(a), at mutation rates beyond 0.01, average adaptiveness 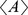 decreased as 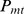 increased, while the fitness value after evolution decreased only slightly. Just below this threshold mutation rate, 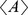 reached a value close to unity and gradually decreased with further decreases in the mutation rate. This suggests existence of an error threshold [25], beyond which a gene expression network with a high 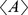 value loses robustness to mutations. To confirm this result, we also computed the change in fitness when a single path in the network was removed from the fitted network with a given 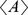 value. As shown in Figure 11, networks with larger 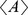 values are less robust to such perturbations. We also carried out numerical simulations with noise in the selection procedure, where networks with lower fitness could leave the offspring with a certain probability. We confirmed that networks with cooperative adaptive responses could evolve when the noise in the selection procedure was sufficiently small, but as this selection noise level was larger, the frequency of cooperative adaptive response decreased.

Since cooperative adaptive response needs a large number of genes, a small mutation rate for the error threshold may be a natural outcome. Such cooperative adaptive response requires mutual cooperative regulation by all other genes. Hence, each gene plays an important role in the regulation of other genes and so their robustness to mutations is weaker. In addition, networks with higher average adaptiveness (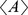) are rather rare. We checked the distribution of 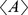 values in randomly generated networks with large fitness values to study the relationships between fitness and 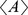 values. Most networks randomly generated showed 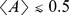, and the probability of the appearance of cooperative adaptive networks was too low when sampling over random networks. The distribution of 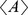 was only slightly shifted towards higher values with an increase in the fitness (Figure S3).

We also examined the robustness of these networks to noise in gene expression dynamics. We added a Gaussian white noise term into eq.(1), with amplitude 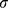 (i.e., to simulate a Langevin equation), and evolved the network to a high fitness value. As shown in Figure 10(b), increases in the noise level caused decreases in the evolved 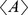 value to 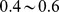. We also examined the decrease in fitness by adding noise to the evolved networks without noise. The fitness decreased when adding noise (of the level 0.05). Fitness was maximal at around 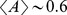, and the decrease due to noise was larger for networks with 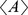 values larger or smaller than 0.6 (Figure 11). Networks with moderate cooperativity (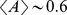) showed higher robustness to noise. In summary, such networks with moderate average adaptiveness achieved higher fitness and robustness to mutations or noise.

### Parameter dependence

We also studied the dependence of the cooperative adaptive response on the parameters in our model system. As for the input values, we set 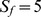 and 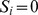 so far. Networks selected with the given fitness under this input condition, indeed could keep cooperative adaptive response for other values of 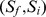 as long as 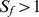 and 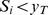 (Figure S4). We also carried out numerical evolution by using different values in 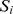 and 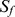, and confirmed that as long as 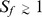, evolved networks achieving large fitness values have large average adaptiveness 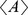.

On another front, although the frequency of cooperative adaptive response only slightly depended on the threshold value, 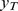, it definitely depended on the parameter 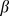, a measure of the sensitivity of gene expression, which corresponds to the Hill coefficient (Figure S5). A high fitness value with cooperative adaptive response (large 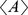) was realized when 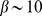. Fitness value decreased with the decrease of 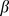, while it kept the highest value with 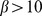. The average adaptiveness 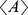 remained large for smaller 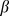, while it showed a drop as 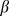 was larger than 20.

Thus, cooperative adaptive response is more important for a system with small 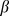 values to achieve a high fitness value. In other words, if the on-off expression is sloppier with a smaller Hill coefficient, perfect adaptation for the target gene cannot be achieved with motifs with a small number of genes, but cooperative adaptive response enables perfect adaptation of target gene expression. Cooperative adaptive response overcomes the sloppiness in gene expression dynamics.

On the other hand, the observation that 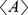 remained at values 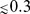 even after 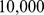 generations of evolution for 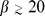 implies that (highly) cooperative adaptive responses did not evolve there. There are two reasons for this. First, by using the time delay strategy, perfect adaptation of the target gene was realized. Recall that in Figure 3 with 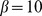, relatively high fitness values were achieved, even with small 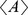 values, using the time delay strategy for earlier generations, and later, highest fitness values were achieved with increased 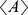 values. For larger 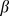 values, however, the fitness value determined by the time delay strategy was close to the highest value, and the gain by the cooperative adaptive strategy was smaller. Second, it was more difficult to show partial adaptations under large 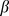 values because 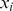 could hardly take intermediate values between 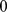 (off) and 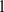 (on) at the steady state due to the step-function nature of the expression. Hence, partial adaptations with distributed values of final gene expression levels, which are required for the cooperative adaptive response, were more difficult to realize with larger 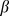 values.

## Discussion

Here, we studied the adaptive response in gene expression dynamics with many degrees of freedom and mutual regulation among genes. We evolved the gene regulatory network using a fitness function for the adaptive response of one target gene. However, we found that evolution led to adaptive expression dynamics in several genes besides the target; this is termed the cooperative adaptive response. Genes that are neither the input nor the target exhibited this adaptive response through mutual regulation.

Furthermore, such networks strengthen the adaptive response of the target gene, even though they are very rare among all possible configurations. However, a sufficient number of genes are required for this cooperative response to occur; small networks with 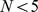 could not evolve at all, but networks with 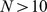 evolved high fitness under all conditions tested. Such singular networks evolved through three steps. First, we obtained an adaptive network utilizing a well-known few-gene network motif, which took advantage of timing differences between target-activating and target-inhibiting signals. Genes that were neither the input nor the target just showed a monotonic change. Next, genes activating the target began to show adaptive responses. At the final stage, genes inhibiting the target also showed adaptive responses. It is worth pointing out that in a network with cooperative responses at this final stage, almost all genes either activate or inhibit the target gene, and the former show adaptive responses with initial up-regulation and subsequent down-regulation, whereas the latter show the opposite adaptive response. Such adaptive responses of most genes are not stipulated by the fitness condition itself, but rather stem from evolution.

As cooperative adaptive responses require a large number of genes, they are not necessarily robust against a large number of mutations. Here, we found that as the mutation rate increased, the number of genes with adaptive responses decreased. Still, for relatively large mutation rates, about half the genes still showed adaptive responses. We also studied the influence of noise and found that networks showing moderate cooperative adaptive responses were able to maintain high fitness values. Conversely, under sufficiently large noise, networks with about half the genes showing adaptive response evolved.

The cooperative adaptive response is more important as the sensitivity in the expression (that corresponds to the Hill coefficient) is lower. In other words, when the on/off expression is sloppier, it is more difficult to achieve higher fitness, i.e., perfect adaptive response, by networks with a few genes. Here, cooperative adaptive response by many genes compensates the sloppiness of each expression dynamics as a collective behavior of many genes, which may be reminiscent of von Neumann's study on reliable computation by unreliable components [26].

As for the adaptive response itself, models with few degrees of freedom have been extensively studied. Ma et al. studied all possible three-node network topologies and found a few minimum adaptive motifs that followed the timing difference observed in the first stage in our simulation [2]. Interestingly, in the cooperative adaptive response observed in the present study, a rather different mechanism was adopted. Indeed, the frequencies of such minimum adaptive motifs decreased as the degree of cooperative response increased, even reaching zero on occasion. Moreover, even when the minimum motifs were included, their behaviors were different from that in isolation; genes that intervene input and output genes showed adaptive responses rather than monotonic responses as in standard motifs. In cooperative adaptive networks, almost all genes except for the input gene showed adaptive responses. Many of them did not show perfect adaptation. Rather, they showed partial adaptation with distributed values of deviation between the initial and final states. Appropriate distribution of such deviations are necessary to achieve adaptive response with mutual activation and inhibition.

Several studies on the responses of cells have shown that many genes exhibit adaptive responses, either initial up-regulation followed by subsequent down-regulation or otherwise, as observed here. Even though the adaptive response is not perfect, many genes (i.e., 50%–70%) show at least partial adaptation, and few genes exhibit monotonic responses or no response [15], [16], [27]. For example, more than half the number of genes in yeast exhibit adaptive responses to several stimuli, as identified by microarray analysis for gene expression patterns [12], [13].

Our study suggested that such responses can generally evolve through gene expression dynamics with mutual regulation of many genes to achieve better adaptive responses of a single gene to environmental changes. It goes without saying that cooperative adaptive responses are robust to replacement of the target gene, because almost all genes already show adaptive responses. We also confirmed that the network can react rapidly to changes in the input gene. Therefore, a network with cooperative responses is advantageous in responding to various types of inputs.

Although our gene expression dynamics and fitness conditions are very simplified relative to the actual biological system, we may expect that the cooperative adaptation dynamics observed in the study can be generalized for systems consisting of a large number of proteins that mutually activate and suppress each other. To confirm this generality, we also simulated models with distributed parameters of the threshold 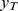 or continuous values of 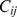 in 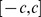 with 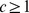, and again found the same cooperative adaptive behavior.

It is often very important and useful to extract motifs with few degrees of freedom from a complicated network by identifying functional roles for such motifs [9], [10]. However, biological networks involve many degrees of freedom. Even if such simple motifs exist, it does not necessarily mean that they function in isolation. Moreover, there may be some other basic mechanism for adaptation inherent in systems with many degrees of freedom. Thus, it is important to study the dynamics and functions of complex networks without decomposing them into motifs with few degrees of freedom. Cooperative adaptive responses are outcomes that emerge only in a system with many degrees of freedom. This may be seen as a kind of cooperative phenomenon, where the adaptive response of one gene relies on that of other genes. Most genes that show up-down adaptive regulation receive positive inputs from up-down adaptive responses and negative inputs from down-up adaptive responses; those that show down-up responses have the opposite interactions. Each gene shows an adaptive response as a result of the adaptive responses of other genes. Thus, adaptive responses are generated in a ‘self-consistent’ manner, through positive and negative adaptive ‘mean-field’ dynamics, generated as a result of the adaptive response of each gene. As discussed in the present model, this self-consistent adaptation is not possible if all genes show perfect adaptation. Instead, most genes show partial adaptation, i.e., final expression levels are not equal to the original levels. Indeed, self-consistent adaptive dynamics over many genes are achieved by suitable distribution of these shifts. Possible condition for such distribution to achieve cooperative adaptive response should be clarified in future, by establishing a proper mean-field analysis. Here, it is interesting to recall that such partial adaptation of gene expression over many components with appropriate distributions is observed in gene expression profiles of yeast *Saccharomyces cerevisiae* [16] and in recent model simulations [27].

In general, it will be important to explore the cooperative dynamics of a network with many degrees of freedom without decomposing it into functional motifs with few degrees of freedom. According to our numerical study, cooperative behaviors are acquired naturally though the evolutionary process in systems with sufficient degrees of freedom. In a system with many genes, there can be some inherent dynamics that are not reducible to a summation of the dynamics of simple motifs. Living cells involve many degrees of freedom that are not necessarily decomposable, and so the search for cooperative dynamics as explored here will be important.

## Supporting Information

Figure S1
**Dependence of the fitness and the average adaptiveness of evolved networks upon network size.** (a) The average fitness and (b) the average adaptiveness values (ordinate) of the evolved networks are plotted against the network size 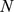 (abscissa) for different 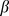, i.e., the sensitivity of the expression of each gene. When the sensitivity of each gene expression is high (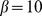 red 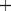), all networks with 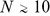 can achieve large fitness and large average adaptiveness values, while the fitness and average adaptiveness values of smaller networks drop drastically with the decrease in 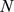 when the sensitivity parameter is lower. Only large networks can keep high fitness and average adaptiveness when the sensitivity of the expression of each gene is lower. (c) The average fitness values (ordinate) of the mutated networks are plotted against the network size 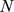 (abscissa). We first prepared 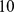 networks for each 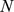 with the largest fitness at the 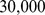
th generation from different strains evolved to satisfy 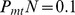 with 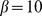. For each network, we then removed a single path connecting any two genes and computed the fitness values for such emulated networks, over all possible removals of single paths. After averaging all possible removals, the average fitness value was obtained. Larger networks showed high fitness even after mutations(PDF)

Figure S2
**Probability of the occurrence of each network motif shown below the graph.** Networks satisfying fitness 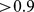 from 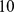 different trials with 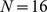 and 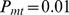 were used. Feed-forward-loop (FFL), negative feedback loop (NFB), and their combination (COM)-type network motifs were the minimum adaptive motifs, whereas P1 and P2 were not adaptive motifs, but had the characteristic of a cooperative network. The dashed line shows the value in the case of a random network. As shown, the negative feedback loop remained at a lower level than random sampling, and there was no clear salient dependence on average adaptiveness defined by 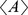. The schematics below the graph illustrate networks with cooperative responses and each network motif. Arrows with solid red lines indicate activation, and arrows with broken blue lines indicate inhibition.(PDF)

Figure S3
**Distribution of average adaptiveness in randomly generated networks.** Occurrence frequency of networks (ordinate) with each average adaptiveness value 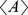 (abscissa) among 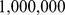 randomly generated networks. The abscissa is divided into 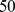 portions. The networks satisfying fitness 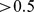 (red plus), fitness 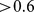 (green cross), fitness 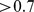 (gray open square), fitness 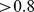 (blue asterisk), and fitness 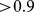 (magenta filled square) are shown.(PDF)

Figure S4
**Dependence of fitness and average adaptiveness on the external input.** Changes in fitness (upper) and average adaptiveness values (lower) according to the external signal 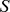 when it showed a stepwise change from 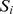 (abscissa) toward 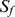 (ordinate) at 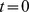. Networks satisfying fitness 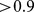 and 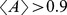 from 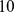 different trials with 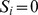, 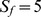, 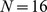 and 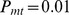 were used. Cooperative adaptive response can be realized when 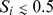 amd 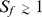.(PDF)

Figure S5
**Dependence of fitness and average adaptiveness on the sensitivity parameter.** Changes in fitness and average adaptiveness (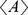) values (ordinate) according to 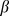 (abscissa) represent the sensitivity of the expression of each gene. Fitness (blue asterisk) and average adaptiveness values (red cross) were obtained from networks evolved following the manner described in the main text, where 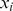 was set at 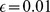 initially and then relaxed to a stationary state under 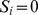 before 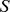 was switched. On the other hand, fitness (cyan square) and average adaptiveness value (gray plus) were obtained from networks evolved with a changed initial state of 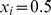 for all 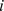 initially before reaching the stationary state. In networks showing cooperative adaptive responses, genes were differentiated into target-activating genes with upward adaptive responses starting from 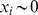 and target-inhibiting genes with downward adaptive responses starting from 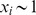. Through the adaptive ‘mean-field’ dynamics generated by these two groups, the adaptive response of the target gene was realized. Here, target-inhibiting genes starting 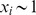 can exist in evolved networks as the interaction term in eq.(1) can activate expression even when 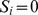, i.e., 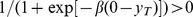 with 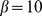 and 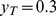. One possible reason to explain why target-inhibiting genes cannot emerge for larger 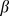 values could be that the stationary state at 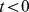 is strongly confined to 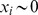 because 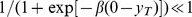. Indeed, the dramatic drop in average adaptiveness (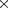) around 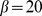 occurred because of this constraint. To remove this influence and to allow for the existence of target-inhibiting genes, we thus changed the initial state to 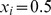 and evolved networks with the same fitness function and parameters as given by 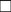 and 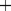. The decrease in the index for the cooperative adaptive response 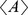 with 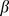 was more gradual compared with the original case, but 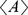 eventually reached 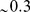 at 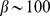. This disappearance of the cooperative adaptive response at sufficiently large 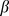 occurred because each gene can assume an off-state (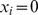) or on-state (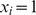) only, so that partial adaptations with intermediate values of final expression levels, which are required for the cooperative adaptive response, were not possible. Values for the network with the largest fitness at the 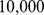
th generation are shown for 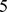 different strains for each 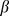. 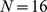, 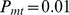, and 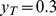.(PDF)
