# Supplementary figures and images for: Cooperative Adaptive Responses in Gene Regulatory Networks with Many Degrees of Freedom
Source: PLoS Comput Biol. 2013 Apr 4;9(4):e1003001. doi: 10.1371/journal.pcbi.1003001 (PMC3616990; doi:10.1371/journal.pcbi.1003001)

Figure S1

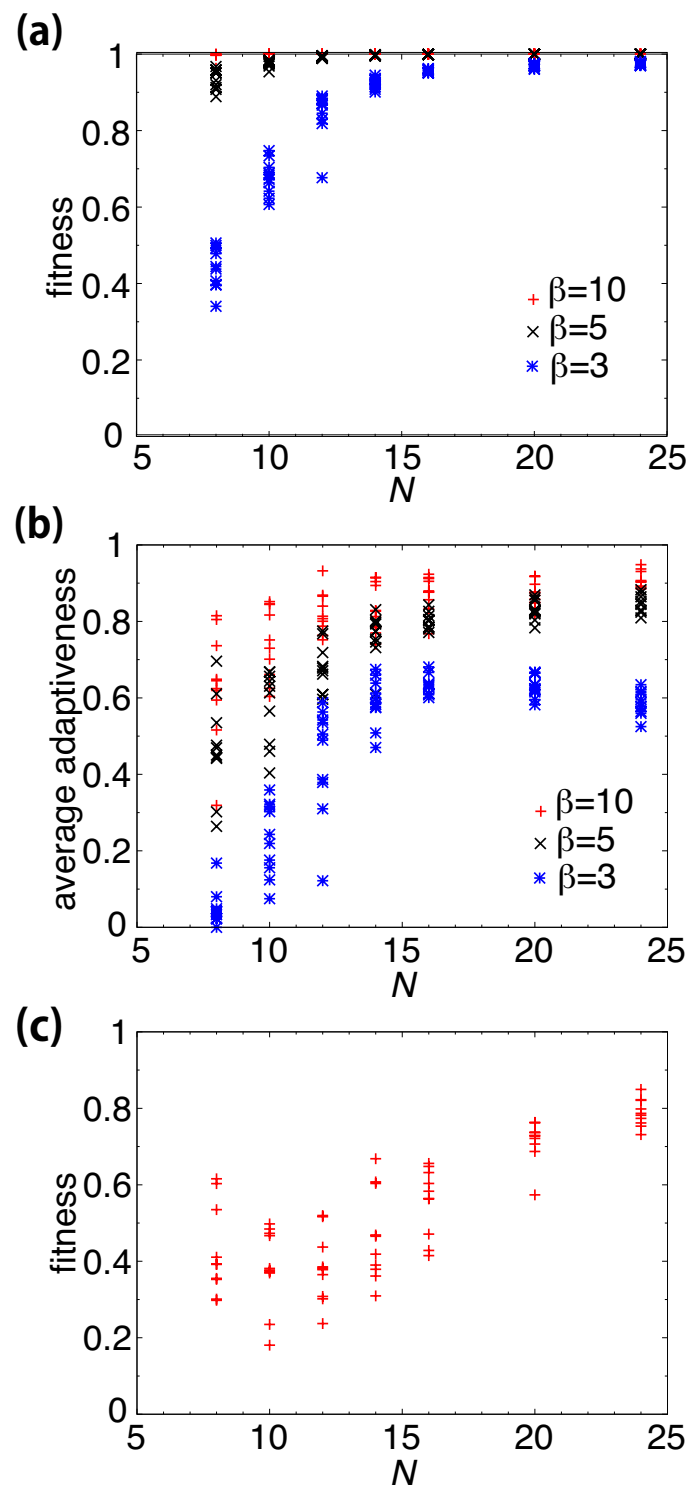

Supplement: Figure S1 — Dependence of the fitness and the average adaptiveness of evolved networks upon network size. (a) The average fitness and (b) the average adaptiveness values (ordinate) of the evolved networks are plotted against the network size (abscissa) for different , i.e., the sensitivity of the expression of each gene. When the sensitivity of each gene expression is high ( red ), all networks with can achieve large fitness and large average adaptiveness values, while the fitness and average adaptiveness values of smaller networks drop drastically with the decrease in when the sensitivity parameter is lower. Only large networks can keep high fitness and average adaptiveness when the sensitivity of the expression of each gene is lower. (c) The average fitness values (ordinate) of the mutated networks are plotted against the network size (abscissa). We first prepared networks for each with the largest fitness at the th generation from different strains evolved to satisfy with . For each network, we then removed a single path connecting any two genes and computed the fitness values for such emulated networks, over all possible removals of single paths. After averaging all possible removals, the average fitness value was obtained. Larger networks showed high fitness even after mutations (PDF) [file pcbi.1003001.s001.pdf]

Figure S2

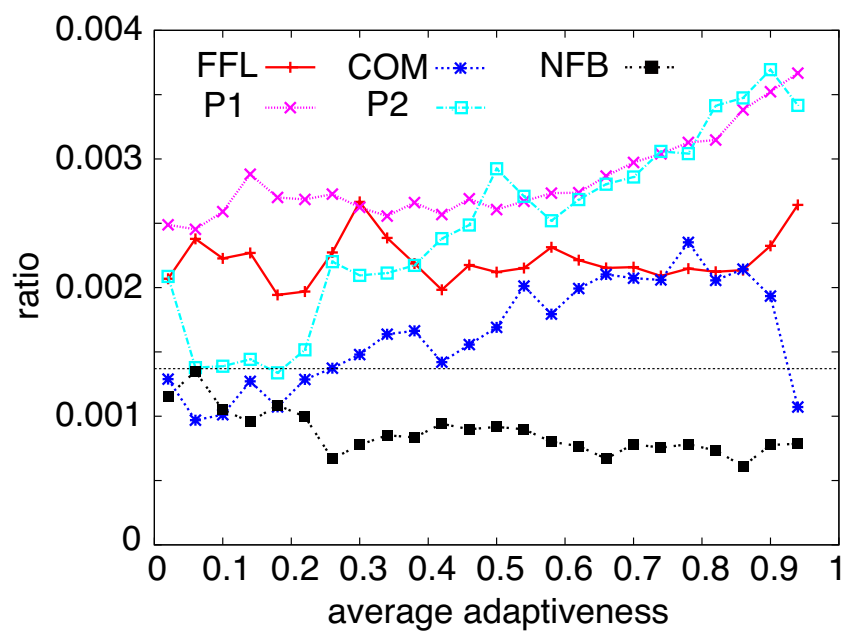

cooperative network

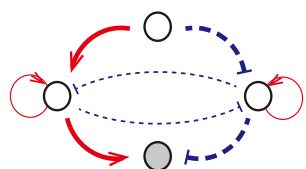

FFL (—+)

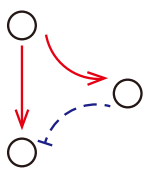

P1 (···x)

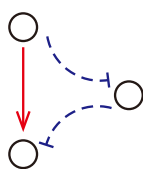

COM (···\*)

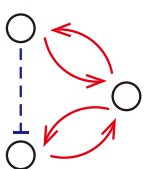

P2 (---□)

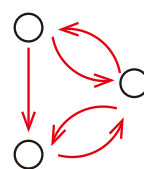

NFB (---■)

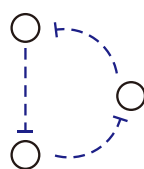

Supplement: Figure S2 — Probability of the occurrence of each network motif shown below the graph. Networks satisfying fitness from different trials with and were used. Feed-forward-loop (FFL), negative feedback loop (NFB), and their combination (COM)-type network motifs were the minimum adaptive motifs, whereas P1 and P2 were not adaptive motifs, but had the characteristic of a cooperative network. The dashed line shows the value in the case of a random network. As shown, the negative feedback loop remained at a lower level than random sampling, and there was no clear salient dependence on average adaptiveness defined by . The schematics below the graph illustrate networks with cooperative responses and each network motif. Arrows with solid red lines indicate activation, and arrows with broken blue lines indicate inhibition. (PDF) [file pcbi.1003001.s002.pdf]

Figure S3

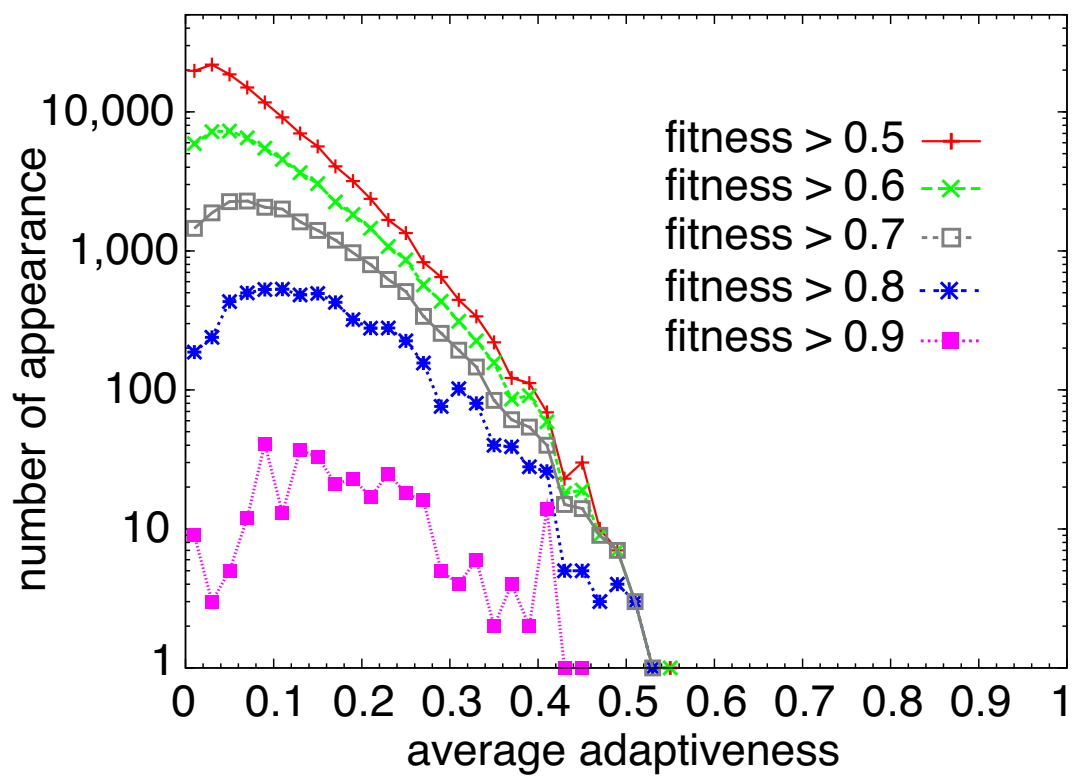

Supplement: Figure S3 — Distribution of average adaptiveness in randomly generated networks. Occurrence frequency of networks (ordinate) with each average adaptiveness value (abscissa) among randomly generated networks. The abscissa is divided into portions. The networks satisfying fitness (red plus), fitness (green cross), fitness (gray open square), fitness (blue asterisk), and fitness (magenta filled square) are shown. (PDF) [file pcbi.1003001.s003.pdf]

Figure S4

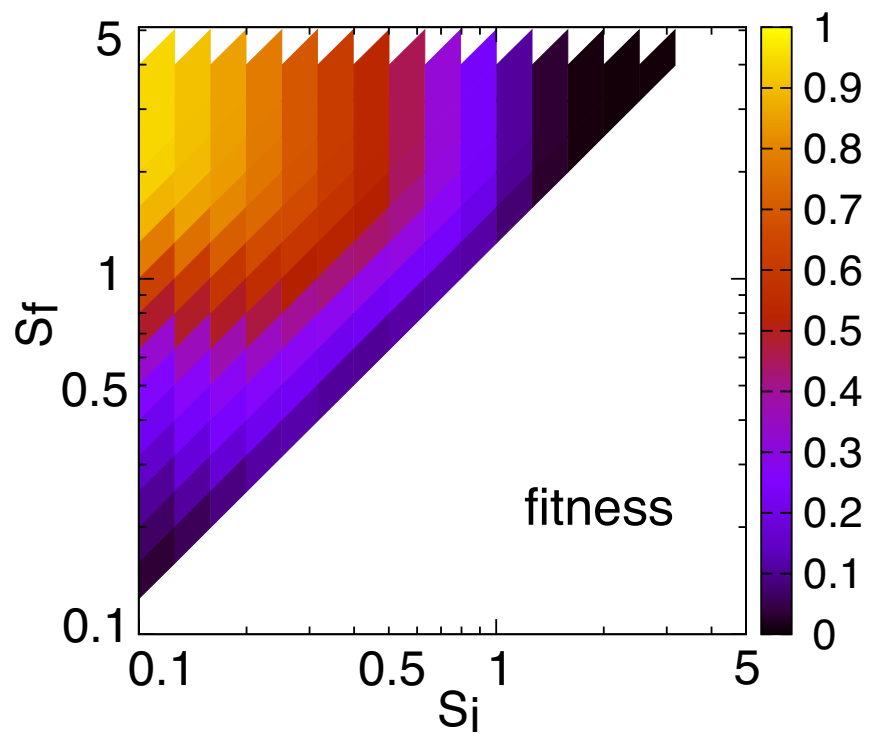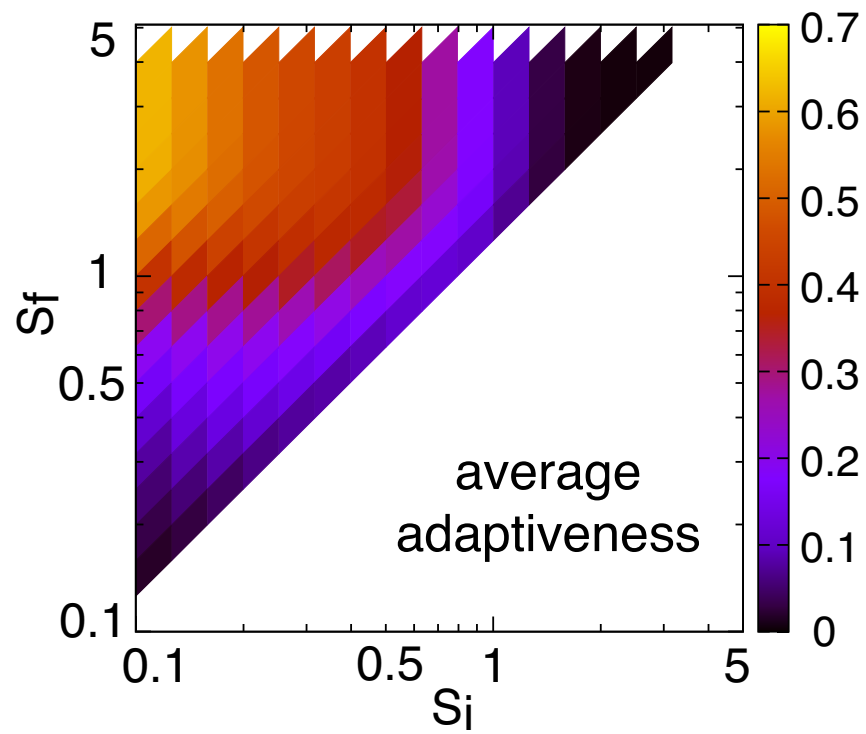

Supplement: Figure S4 — Dependence of fitness and average adaptiveness on the external input. Changes in fitness (upper) and average adaptiveness values (lower) according to the external signal when it showed a stepwise change from (abscissa) toward (ordinate) at . Networks satisfying fitness and from different trials with , , and were used. Cooperative adaptive response can be realized when amd . (PDF) [file pcbi.1003001.s004.pdf]

Figure S5

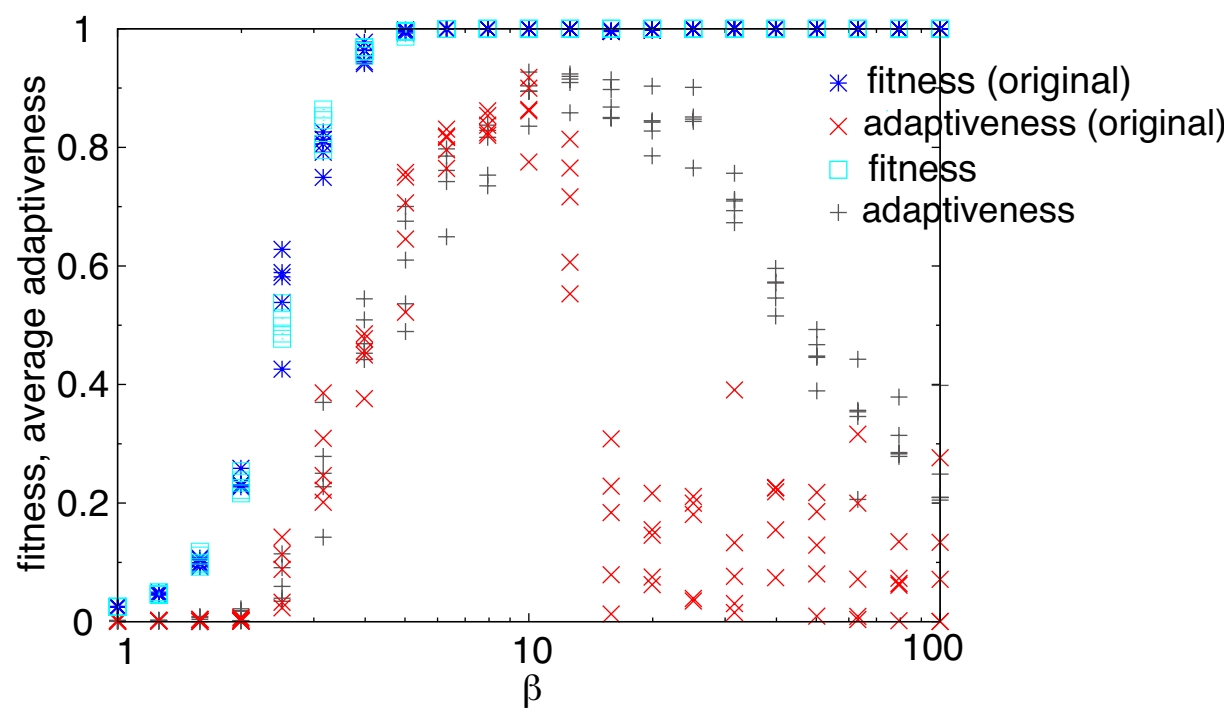

Supplement: Figure S5 — Dependence of fitness and average adaptiveness on the sensitivity parameter. Changes in fitness and average adaptiveness () values (ordinate) according to (abscissa) represent the sensitivity of the expression of each gene. Fitness (blue asterisk) and average adaptiveness values (red cross) were obtained from networks evolved following the manner described in the main text, where was set at initially and then relaxed to a stationary state under before was switched. On the other hand, fitness (cyan square) and average adaptiveness value (gray plus) were obtained from networks evolved with a changed initial state of for all initially before reaching the stationary state. In networks showing cooperative adaptive responses, genes were differentiated into target-activating genes with upward adaptive responses starting from and target-inhibiting genes with downward adaptive responses starting from . Through the adaptive ‘mean-field’ dynamics generated by these two groups, the adaptive response of the target gene was realized. Here, target-inhibiting genes starting can exist in evolved networks as the interaction term in eq.(1) can activate expression even when , i.e., with and . One possible reason to explain why target-inhibiting genes cannot emerge for larger values could be that the stationary state at is strongly confined to because . Indeed, the dramatic drop in average adaptiveness () around occurred because of this constraint. To remove this influence and to allow for the existence of target-inhibiting genes, we thus changed the initial state to and evolved networks with the same fitness function and parameters as given by and . The decrease in the index for the cooperative adaptive response with was more gradual compared with the original case, but eventually reached at . This disappearance of the cooperative adaptive response at sufficiently large occurred because each gene can assume an off-state () or on-state () only, so that partial adaptations with intermedi [file pcbi.1003001.s005.pdf]
